# Ageing, Shocks and Wear Mechanisms in ZTA and the Long-Term Performance of Hip Joint Materials

**DOI:** 10.3390/ma10060569

**Published:** 2017-05-24

**Authors:** Armelle Perrichon, Bernard Haochih Liu, Jérôme Chevalier, Laurent Gremillard, Bruno Reynard, Frédéric Farizon, Jiunn-Der Liao, Jean Geringer

**Affiliations:** 1 CIS-EMSE, SAINBIOSE, INSERM U1059, Ecole Nationale Supérieure des Mines de Saint-Etienne, F-42023 Saint-Etienne, Univ Lyon, F-69,000 Lyon, France; perrichon.armelle@gmail.com; 2 Department of Materials Science and Engineering, National Cheng Kung University, Tainan 701, Taiwan; hcliu@mail.ncku.edu.tw (B.H.L.); jdliao@mail.ncku.edu.tw (J.-D.L.); 3 MATEIS, UMR CNRS 5510, Univ Lyon, INSA-Lyon, 20 Avenue Einstein, 69621 Villeurbanne, France; jerome.chevalier@insa-lyon.fr (J.C.); laurent.gremillard@insa-lyon.fr (L.G.); 4 Laboratoire de Géologie de Lyon, UMR CNRS 5276, Univ Lyon, Ecole Normale Supérieure de Lyon, Université Claude Bernard Lyon 1, 46 Allée d’Italie, 69364 Lyon Cedex 07, France; bruno.reynard@ens-lyon.fr; 5 Chirurgie Orthopédique et Traumatologie, CHU Saint-Etienne, Avenue Albert Raimond, 42270 Saint-Priest-en-Jarez, France; frederic.farizon@chu-st-etienne.fr

**Keywords:** ceramics, shocks, wear, hip implants, nanoindentation, zirconia phase transformation

## Abstract

The surface morphologies and microstructures of Zirconia Toughened Alumina (ZTA) femoral heads were analyzed following in vitro tests aiming to simulate in vivo degradation. Three phenomena potentially leading to degradation were investigated: shocks, friction and hydrothermal ageing. Shocks due to micro-separation created the main damage with the formation of wear stripes on the femoral head surfaces. Atomic Force Microscopy (AFM) images suggested the release of wear debris of various shapes and sizes through inter- and intra-granular cracks; some debris may have a size lower than 100 nm. A decrease in hardness and Young’s modulus was measured within the wear stripes by nanoindentation technique and was attributed to the presence of surface and sub-surface micro-cracks. Such micro-cracks mechanically triggered the zirconia phase transformation in those worn areas, which in return presumably reduced further crack propagation. In comparison with shocks, friction caused little wear degradation as observed from AFM images by scarce pullout of grains. The long-term resistance of the ZTA composite material against hydrothermal ageing is confirmed by the present observations.

## 1. Introduction

Wear is one of the most important factors limiting the longevity of a total hip prosthesis. It is of great importance because the wear debris released in the human body may trigger osteolysis and aseptic loosening [1], a possible cause of pain, reduced mobility, and the need for revision surgeries. Ceramics are materials of choice for hip joint bearings due to their excellent biocompatibility, mechanical and corrosion resistance. Ceramic-on-Ceramic (CoC) bearings also exhibit a superior wear performance compared to alternative couples such as Metal-on-Metal (MoM), Metal-on-Polymer (MoP) or Ceramic-on-Polymer (CoP) [2,3], which leads to a decrease amount of debris [4].

Introduced in the 1970s [5], alumina (Al_2_O_3_) was the first ceramic widely dedicated to orthopedics applications. It exhibits low friction, good wettability and wear resistance but the first generations of alumina bearings experienced high fracture rates [6]. Today’s failure rate with alumina bearings is very low, but the modest toughness of the material limits its use in challenging situations. Zirconia (ZrO_2_) was introduced in the 1980s to overcome this problem thanks to the improved fracture toughness and mechanical strength compared to alumina. Zirconia exhibits three crystalline structures (monoclinic, tetragonal, and cubic) and has the greatest mechanical resistance in the form of tetragonal phase, which is metastable at room temperature and leads to phase transformation toughening. Thus it is manufactured with the addition of oxides, mainly yttria, calcia or ceria, which act as stabilizers for the tetragonal phase. Tetragonal zirconia polycrystals doped with yttria (Y-TZP) have been the most commercialized materials [7].

The development of zirconia-based hip joints however encountered a drastic slow down after the catastrophic failure of specific batches of Y-TZP heads processed by the major company providing the material in the early 2000s. This phenomenon was attributed to a change in the manufacturing process, which led to the premature Low Temperature Degradation (LTD) or ageing of the material in vivo accompanied by a phase transformation from tetragonal to monoclinic phase [8]. This martensitic type phase transformation is promoted by hydrothermal conditions (i.e., LTD in aqueous environment like the human body) and mechanical stresses. With 16% shear and 4% volume expansion [9], the phase transformation is the origin of the high fracture toughness, i.e., resistance to crack propagation of zirconia based materials. Toughening mechanisms involve the dissipation of the energy associated with the crack propagation in the stress-induced transformation process at the front of a crack tip and in overcoming the compression stresses resulting from the volume expansion. However, this advantage is lost once the material undergoes spontaneous phase transformation at the surface in the presence of water. This phenomenon referred as LTD leads to surface damage, e.g., grains pullout or pop-out, and might be at the origin of subsequent wear in bearings [8,9,10]. Micro-cracking at grain boundaries may be produced as a result of large shear strain associated with martensitic plate formation during the transformation [11].

The best balance between hardness, toughness and hydrothermal resistance was targeted thanks to alumina–zirconia composites in order to improve the ceramic properties. The combination of both materials allows improving the mechanical strength and toughness compared to monolithic alumina while avoiding the occurrence of LTD as encountered in Y-TZP [12]. Zirconia toughened alumina (ZTA) composites have been mainly developed and commercialized in the last fifteen years [13]. Their good strength mainly originates from a fine microstructure that includes well dispersed and isolated zirconia grains constrained in an alumina matrix.

In order to predict the long-term materials performance in the conditions of use, accelerated in vitro experiments are required and must reproduce as close as possible in vivo conditions, which include wear, micro-separation and physical ageing of the material in the presence of body fluids. In previous works [14,15], we experimentally studied the effects of three sources of wear and ageing damage on ZTA hip joints: friction, hydrothermal ageing, and shocks. Shocks are associated with micro-separation i.e., the occurrence of a separation between the femoral head and cup that induces short and high contact stresses between those two components at heel-strike [16,17]. In the experiments, shocks were simulated on a specific shock device [18]; friction was reproduced on a standard hip-walking simulator, and accelerated hydrothermal ageing tests were conducted in an autoclave. The experiments and a comparison with wear patterns on retrieved implants (clinical cases [14,15])made of ZTA revealed that shocks are at the origin of the main wear damage with the formation of wear stripes on the femoral head surfaces, and that they have to be considered to reproduce the in vivo degradation.

Following this previous work, where we were able to simulate and reproduce degradation mechanisms observed in ZTA implants, the main objective of the current work was to describe the microstructural features of damage after friction, shocks or hydrothermal ageing. Atomic Force Microscope (AFM) and Dual-Beam Focused Ion Beam (DB-FIB) were used to reveal the damage mechanisms on and below the bearing surfaces. The mechanical properties were furthermore determined by nanoindentation in order to evaluate their evolution in worn and aged areas of the damaged implants. Through the comparison of the different degradation mechanisms, we aimed at understanding the long-term potential risks in vivo for ZTA hip implants.

## 2. Materials and Methods

### 2.1. Materials

**Figure 1:**
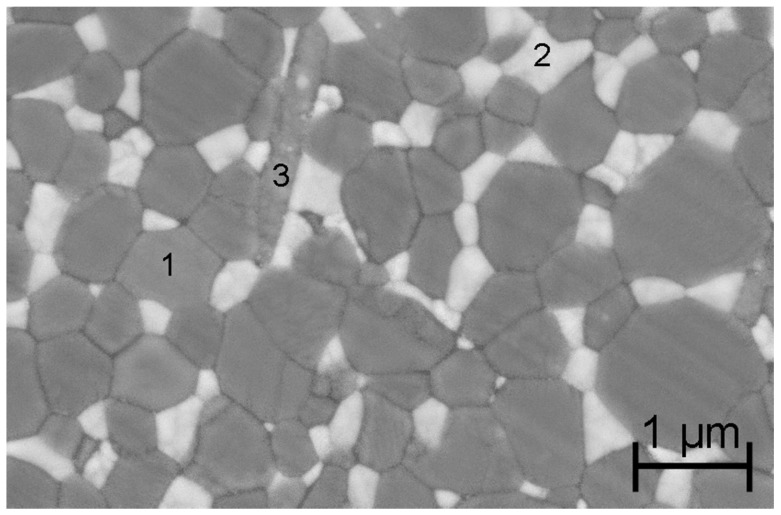
Scanning Electron Microscopy (SEM) image of a thermally etched as-received Biolox^®^ delta implant: (1) alumina grain; (2) yttria-stabilized zirconia grain; and (3) platelet-shaped crystal of strontium aluminate.

The studied hip joints (36 mm femoral heads and acetabular cups) are made of ZTA composite belonging to the latest generation of commercialized Biolox^®^ delta implants, CeramTec AG (Plochingen, Germany). SEM imaging (MEB FEG ZEISS SUPRA 55 VP) of a thermally etched (1300 °C in air for 12 min) as-received Biolox® delta implant shows yttria-stabilized zirconia grains homogeneously dispersed in a continuous alumina matrix (Figure 1). These zirconia grains can transform under stress at the vicinity of an advancing crack and are the main source of toughening. Addition of strontium oxide created platelet-shaped crystals of strontium aluminate composite that may induce an additional toughening by deflecting crack growth. The manufacturing process yields a fine microstructure (alumina, roughly 1 μm, and zirconia lower than 0.5 μm of grain size).

The ZTA hip implants were previously submitted to various in vitro experiments and analyses [14,15] as summarized in Table 1. One specimen (*n* = 1) was selected for analysis in each experimental condition. The sample #SIM was submitted to 6 M cycles on a standard hip-walking simulator (858 Mini Bionix II test system, MTS) in anatomical position under the conditions defined by ISO standard 14242-1 [19]. We imposed a vertical mechanical load of 3 kN as well as hip movements (abduction/adduction, flexion/extension, and external/internal rotations) that define a gait cycle. The samples #SH6 and #SH9 experienced 1.5 M short-time shocks of 6 and 9 kN, respectively, under an imposed vertical micro-separation of 1 mm on a specific shock device [18]. The tests on both experimental devices were conducted in a fetal bovine serum solution (Biowest^®^) diluted in desionized water in order to get a similar proteins concentration of 30 g/L to serum in the human body. It was replaced with fresh lubricant every 500,000 cycles in the case of wear tests and 50,000 shocks in the case of shock testing. Long-term hydrothermal ageing in an autoclave (Micro 8, 4001745, autoclave MED8, JP Selecta S.A.) was also performed to accelerate the LTD of the zirconia phase. It consists in a thermal treatment in water-saturated atmosphere able to simulate the degradation that the material suffers for longer times at lower temperature i.e., at the body temperature [12]. One-hour in vitro under controlled hydrothermal conditions of 134 °C and 2 bars is equivalent to a range of exposure time of 2–4 years at 37 °C [20,21]. The sample #AUT was submitted to 360 h of artificial ageing that simulate natural ageing for several hundreds of years in the conditions of use. One as-received implant (#PRIST) was used as the control.

This study focuses on the femoral head component of the hip joint; the analysis of the cup requires the development of further specific characterization procedures due to its concavity. In previous studies [14,15], we applied non-destructive techniques to characterize the head surfaces at several steps of in vitro tests. The occurrence of zirconia phase transformation was quantified by micro-Raman spectroscopy (Horiba JobinYvon HR800, laser λ = 514.5 nm). The monoclinic phase volume content (*V*_m_, %) was calculated based on the intensities of four characteristic peaks used to distinguish zirconia polymorphs and Clarke and Adar formula [22]. 3D optical profilometry (Bruker nanoscopeTM, ex. Veeco, Wyko NT 9100) was used to evaluate the arithmetic roughness parameter, *S*_a_ (nm); the size of every analyzed area was 0.4 mm × 0.6 mm. Resulting averaged values of *S*_a_ and *V*_m_ for each implant measured at the end of the tests are summarized in Table 1. Full details of the tests, analyses and results can be found in Ref. [14,15].

### 2.2. Characterization Methods

**Figure 2:**
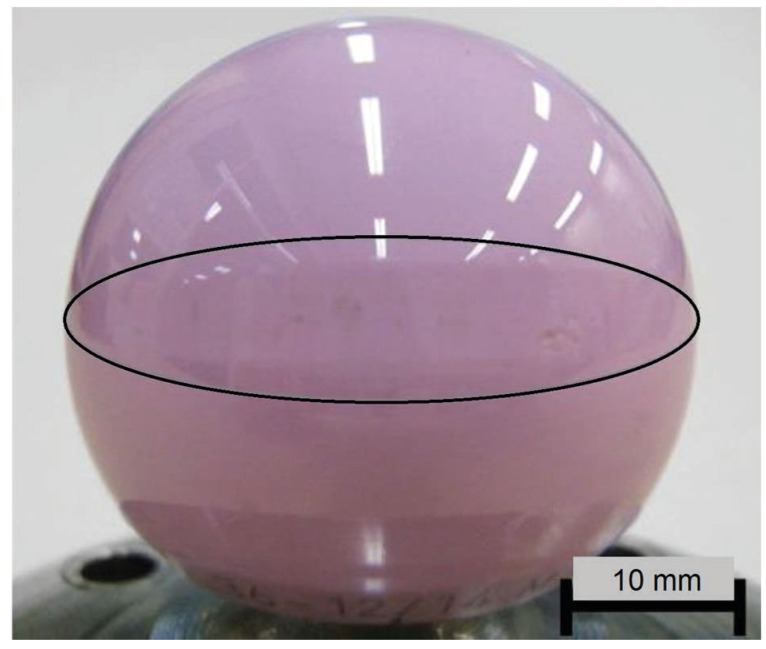
Image of a femoral head with a visible wear stripe (surrounded by an oval shape).

At the end of the in vitro tests, a section of interest was extracted from each head using a linear precision saw (ISOMET 4000, Buehler) to allow further characterization. The sectioning was done well away from the area of interest in order to avoid sawing-induced damage near the cuttings. It is worth noting that the sections of interest in the samples #SH6 and #SH9 (shock tests) exclusively held areas of visible wear stripes, typical of micro-separation (Figure 2).

An AFM (Bruker Dimension ICON, Bruker Corp.) was used to examine the surface morphology and roughness of the femoral heads. It offers the possibility of observing non-conductive bulk materials with a sub-nanometer vertical resolution and nanometer lateral resolution without specific sample preparation, except cleaning of the sample surface. The images were collected in contact mode using silicon nitride probes at room temperature in an ambient atmosphere and were processed using the commercially available software supplied with the microscope system (NanoScope Analysis). The contact mode involves the scanning of a surface with a probe tip at a constant applied force and the measurement of the deflection variation that is directly related to changes in surface relief. Two types of images can be obtained: height image and derivative (or error) image. The first is a topological image related to the height of the surface relief (the higher appears the brighter); the second is related to the gradient of surface relief evolution (the more abrupt change appears the brighter one).

DB-FIB (FEI Nova 200 NanoLab) was used to observe 2D cross-section trenches. A gallium focused ion beam was used at normal incidence to locally cut the sample and the subsurface microstructure was imaged by SEM with a 55° angle between the electron beam and the cross-sectional planes. The sample surfaces were pre-coated with platinum to protect the region of interest from gallium-ion beam damage. Charging effects may affect the stability and resolution of the SEM images of non-coated cross-sectional planes.

Nanoindentation tests were carried out to assess the mechanical properties of the surface and subsurface of the femoral heads. We used a nanoindenter (Nanoindenter G200, MTS, Agilent Technologies, Santa Clara, CA, USA) equipped with a continuous stiffness measurement (CSM) module that allows continuous measurements of load (*P*) and contact stiffness (*S*) as a function of penetration depth (*h*). Hardness (*H*) and Young’s modulus (*E*) were calculated using Oliver and Pharr’s method [23]. The hardness is obtained by dividing the maximum load by the projected contact area under loading:

(1) $$H=\frac{P_{max}}{A\left(h_{c}\right)},$$

where *A*(*h*c) is a calibrated area function based on the contact depth (*h*c) along which contact is made between the indenter and the specimen. It is continuously calculated from the well-known geometry of the indenter through its form factor ε:

(2) $$h_{c}=h_{max}-\varepsilon \frac{P}{S},$$

where *ε* has an empirical value of 0.75 for the employed Berkovich indenter and *h*_max_ is the maximum displacement in depth. *E* is calculated from the effective elastic modulus (*E*_eff_) that takes into account the elastic displacements of both the specimen and the indenter and is defined by:

(3) $$E_{eff}=\frac{1}{\beta }\frac{\sqrt{\pi }}{2}\frac{S}{\sqrt{A\left(h_{c}\right)}},$$

where *β* is a dimensionless parameter equal to 1.034 for a Berkovich indenter.

Nanoindentation techniques involve small load and indenter size that are of great interest for hard ceramics that exhibit a tendency to cracking and chipping. Small load and indenter size also allow measuring mechanical properties at micro and nano scales, on areas of a few μm^2^ so that the curvature of the prosthesis can be neglected. On the femoral head surfaces, indentations were made up to a controlled maximum penetration depth of about 1200 nm and at a constant strain rate of 0.05 s^−1^, which corresponded to a maximum load in the range 530–550 mN. We used a 3-sided pyramid Berkovich indenter calibrated on a single-crystal aluminum. Fifteen independent indentations were performed on each sample and the results were averaged. A surface examination was made by AFM before the nanoindentation tests to make sure that the roughness of the tested areas was below the limit of 5% of the maximum penetration depth as recommended by ISO standard 14577 [23]. *S*_a_ was averaged over five independent AFM images of a size 40 μm × 40 μm; every image was subjected to a second-order flattening in order to remove the relief effect due to the spherical shape of the prosthesis. AFM images were also recorded after the tests to verify the absence of cracking and chipping on the ceramic surfaces and to assess the size of the indents. The Student test at 5% level of significance was used to detect differences among the mechanical properties of the five analyzed samples.

## 3. Results

### 3.1. Surface and Subsurface Damage

**Figure 3:**
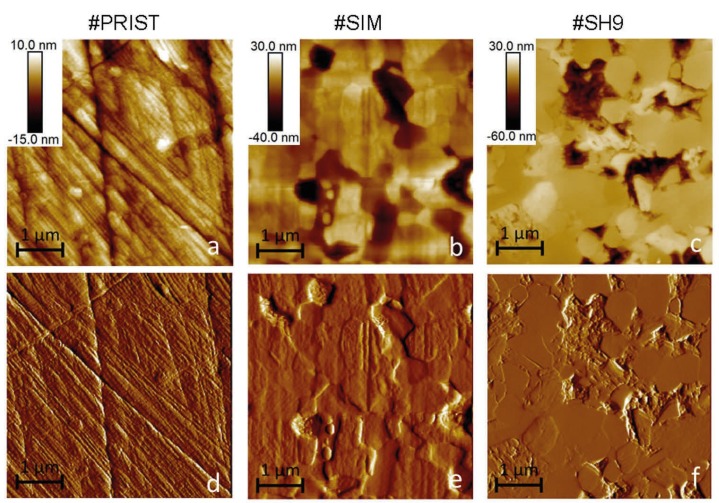
AFM height (top) and derivative (bottom) images of the control-sample #PRIST ((**a**) and (**d**)), the sample #SIM ((**b**) and (**e**)) and the sample #SH9 (wear stripe area, (**c**) and (**f**)).

Polishing scratches dominated the surface of the control-sample (#PRIST, Figure 3a,d) and prevented the detection of its microstructure. The surface of the sample #AUT also revealed polishing marks and only differed from the one of the pristine sample because of a few pullout of grains. The area of pitting reached less than 1.5% of the total imaged areas and Rmax, which represents the maximum vertical distance between the highest and lowest data point in an image, averaged about 170 nm (#AUT) compared to 40 nm for #PRIST.

The surface sample of #SIM was non-homogeneous and the severity of the degradation varied depending on the zones analyzed on the head surface; however we did not find any correlation between the type of damage and the location of the zones on the head. The wear features exhibited on the most damaged zones are shown in Figure 3b,e. The area of pitting reached up to 12% within those zones and Rmax averaged about 170 nm. The polishing features were partly erased on the surface sample depending on the areas of analysis; some areas were similar to the surface of the control-sample.

The surfaces imaged on the sample #SH9 revealed other wear features (Figure 3c,f). They did not contain any initial polishing scratches as the ones observed on the surface of the pristine sample since the areas of interest (areas of wear stripes) have been created through the continuous release of materials (from surface to bulk) during in vitro shock tests [15]. The area of pitting averaged here 12% of the total imaged areas and Rmax was about 300 nm. Micro-cracks were observed at the grain boundaries. The worn surface exhibited smooth and spread grains compared to that observed on the surface sample of #SIM. The surfaces imaged on the sample #SH6 (areas of wear stripes) showed similar wear features as the sample #SH9; Rmax averaged about 300 nm.

**Figure 4:**
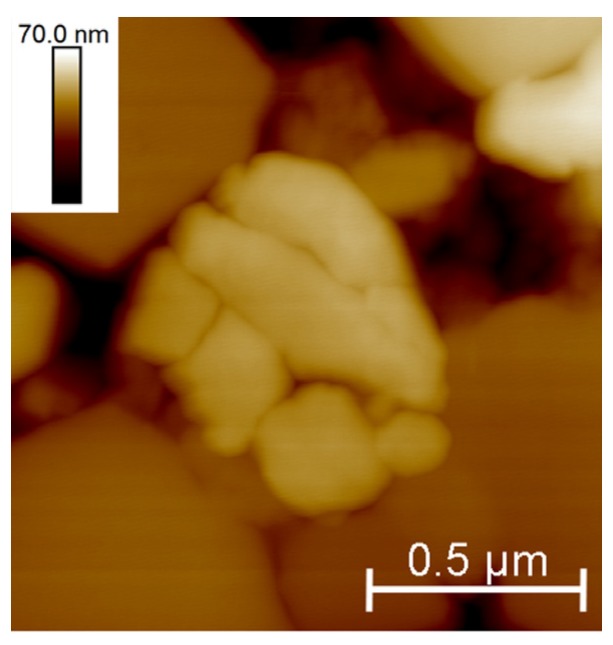
Atomic Force Microscope (AFM) height image of the sample #SH9 illustrating a cracked grain within the wear stripe area.

**Figure 5:**
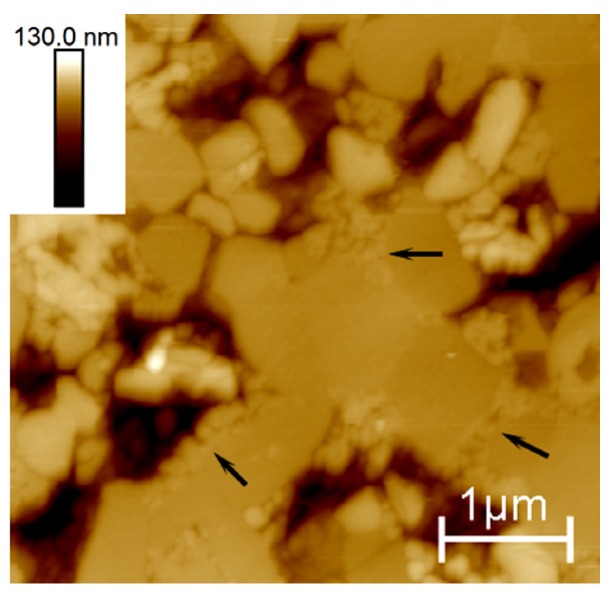
AFM height image of the wear stripe area of the sample #SH9 illustrating debris piled into pits and at the grain boundaries.

A closer inspection of the surfaces revealed the presence of cracked grains (Figure 4) within the wear stripes of the samples submitted to shocks (#SH6 and #SH9). Intra-granular cracks were observed and suggest the formation of wear debris whose size is smaller than the grain size and whose shape is irregular; some debris may have a size lower than 100 nm. Such debris was identified on the worn surface into the remaining pits caused by grains pullout as well as at the grain boundaries (Figure 5). An EDX analysis showed that the chemical composition on the worn areas was not different to the debris-free areas (such as on #AUT or #SIM), which ruled out the possibility of a contamination and confirmed the presence of ceramic debris.

**Figure 6:**
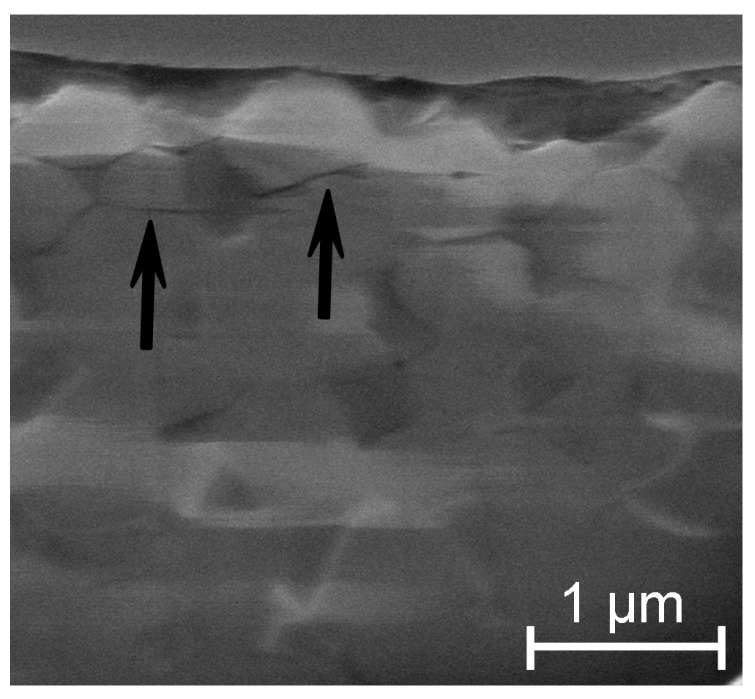
Image from a cross-section obtained by DB-FIB below the worn surface of the sample #SH9; a few micro-cracks were identified (black arrows).

A few micro-cracks were observed by Dual Beam-Focused Ion Beam (DB-FIB) at the subsurface of the sample #SH9, i.e., below the area of wear stripe (Figure 6). The micro-cracks were mainly horizontal and limited to the very first μm in depth. The subsurface microstructure of the control-sample was also imaged for the comparison: no micro-cracks could have been observed in the studied area. The DB-FIB analysis will be more detailed in a next paper.

### 3.2. Mechanical Properties

**Figure 7:**
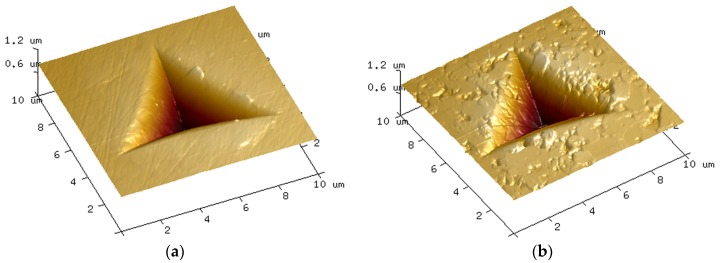
3D AFM images of residual indents on: (**a**) undamaged surface (#PRIST); and (**b**) area of wear stripe (#SH9).

Nanoindentation tests revealed loading-unloading curves without discontinuities such as pop-in or pop-out effects [24] independently of the sample condition. 3D AFM images on undamaged (#PRIST) and damaged, i.e., wear stripe areas, (#SH9) surfaces show the residual triangular indent from the Berkovich indenter (Figure 7). On all the tested samples, the AFM images confirmed that the applied load did not induce the formation of radial cracks around or at the edge of the indent. The size of the indents was determined from these images: the triangle sides averaged 8 μm, i.e., a total area of about 28 μm^2^. The indent size was much higher than the mean grain size, showing that the mechanical measurements were not affected by the grain size distribution in the composite.

**Figure 8:**
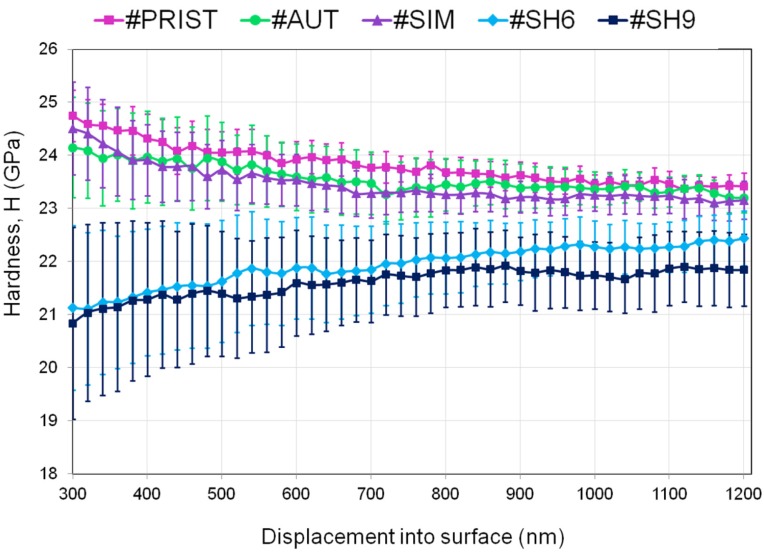
Hardness evolution as a function of penetration depth: averaged curves over the fifteen independent measurements performed on every sample.

**Figure 9:**
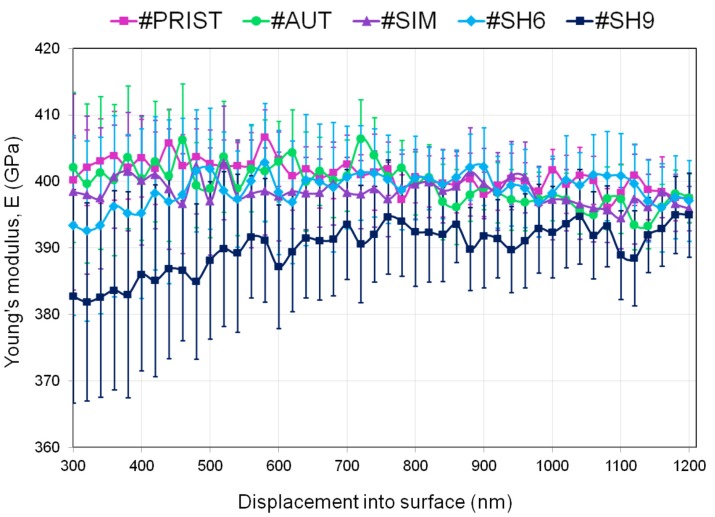
Young’s modulus evolution as a function of penetration depth: averaged curves over the fifteen independent measurements performed on every sample.

Figure 8 and Figure 9 illustrate the averaged data points calculated every 20 nm for hardness (*H*) and Young’s modulus (*E*), respectively. Every point represents the average of the fifteen indents and is accompanied by a 95% confidence interval. The values of hardness of the samples #SH6 and #SH9 were significantly lower than the ones of the samples #PRIST (*p* < 0.001 for #SH9 and *p* = 0.001 for #SH6), #AUT (*p* = 0.002 for #SH9 and *p* = 0.005 for #SH6) and #SIM (*p* = 0.003 for #SH9 and *p* = 0.007 for #SH6). The decrease in hardness (with respect to the control-sample) was higher for #SH9 than for #SH6 and was reduced with the penetration depth (Table 2). The two samples #SH6 and #SH9 exhibited a singular behavior compared with the samples #PRIST, #AUT and #SIM: the former experienced an increase in *H* with increasing depth (Figure 8). During indentation tests, ceramics have a tendency to show a decrease in the mechanical properties (*H* or *E*) as a function of depth due to the indentation size effect (ISE) that may be attributed to several phenomena (elastic recovery, dislocations, work hardening, etc. [25]). The increasing trend observed on the samples #SH6 and #SH9 may be associated with the degree of degradation within the wear stripes (increasing roughness, grains pullout, cracks at grain boundaries, etc.) that must be reduced in the bulk. In terms of Young’s modulus, the sample #SH9 showed lower values compared with the other samples (Figure 9). Its values tended towards those of the control-sample after the first μm in depth (Table 2). For every sample, the curve of *E* was less smooth than the one of *H*.

## 4. Discussion

### 4.1. Ageing, Shocks and Wear Mechanisms of ZTA Hip Joints

The sensitivity to hydrothermal ageing remains one of the main concerns of composite implants containing zirconia. It is at the origin of the zirconia phase transformation that is enhanced in aqueous environment [8]. The extent of the transformation is quantified by the amount of the monoclinic phase and its potential detrimental effects are usually evaluated in terms of surface roughness increase (that affects the wear resistance). Several authors have recently reported the alteration in the mechanical properties of experimentally aged zirconia based ceramics (TZP). The trend is a decrease in *H* and *E* with increasing *V*_m_: up to 20% [26], 30% [24,27,28] and 40% [29] of decrease for the most degraded samples; the decrease in E is most of the time lower than the one in H. Catledge et al. [30] reported a similar evolution on retrieved zirconia implants (TZP) with a decrease in hardness that reached more than 40%. Retrieved implants were submitted in vivo to both hydrothermal and mechanical environment.

**Figure 10:**
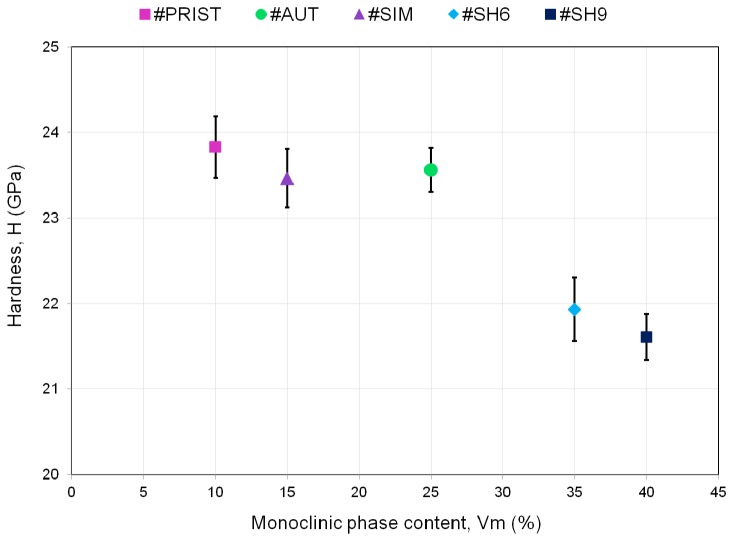
Relation between the occurrence of the phase transformation and the hardness value (averaged over the range of the analyzed penetration depth 300–1200 nm).

There is a lack of available data concerning a decline in the mechanical properties of recent alumina–zirconia composites as a response to hydrothermal alteration. The sample #AUT went here through a heavy and tough treatment in an autoclave that simulates hundreds of years of in vivo time, i.e., that is representative of the material long-term performance. Alteration on the implant surface is characterized by a few grains pullout that represent a small area of pitting (less than 1.5%) compared to that observed on the samples mechanically damaged (#SIM, #SH6 and #SH9). Both H and E are comparable to non-altered control on the sample #AUT whose monoclinic phase content rose from 10% to 25% on the surface (Figure 10). This confirms the long-term resistance of tailored ZTA composite against hydrothermal ageing and the fact that (moderate) increase of monoclinic fraction does not necessarily means decrease in mechanical properties for this specific type of composite.

The hip joint wear resistance is commonly assessed by the standard test on a hip-walking simulator. In our previous study [15], we showed that the wear volume associated with this test is negligible compared with the one produced by shocks but we could not detect any wear features at a macroscopic scale. AFM analysis revealed here at lower scales non-homogeneous wear damage induced by the friction effects applied on the surface of #SIM. Localized damaged areas exhibited pullout of grains (Figure 3). Firstly, this explains the origin of the wear volume release that is the quantitative factor commonly used to evaluate the wear resistance after the standard test. Secondly, this suggests a wear debris size equivalent to the grain size.

The relevance of the single use of hip-walking simulator as a standard wear test has already been discussed [31,32] and the present study must contribute to the discussion. The analysis of retrieved implants [14,15] revealed wear features that the standard test is not able to reproduce contrary to the shocks. Shocks have been identified as the main wear degradation mechanisms symbolized by the formation of wear stripes in which the phase transformation occurs, according with that observed on explants. The decrease in the surface mechanical properties (Figure 10) and the formation of non-homogenous (in terms of size and shape) wear debris (Figure 4 and Figure 5) characterize the alteration of the implant within the wear stripes. The need to introduce several loading conditions associated with different routine activities, i.e., from steady walking conditions on hip-walking simulator to intense activities such as climbing stairs on the shock machine, is essential in order to experimentally reproduce the implant in vivo wear degradation, i.e., from low to severe degradation, respectively.

### 4.2. Long-Term Performance of ZTA Hip Joints

This study aims at better predicting the long-term performance of ZTA hip joints that endure severe hydrothermal and mechanical conditions. Even if performed in simple conditions that limit extrapolation to critical conditions of use, hydrothermal ageing is not the primary origin of degradation, at least when assessed with steam sterilization cycles. Their resistance to shocks must be a key point in the performance because it represents the most important origin of wear debris. The AFM analysis provided here a better understanding of the wear processes within the stripes. The mechanical stresses at shocks induce grain boundaries (i.e., weak interfaces in the microstructure) to crack (Figure 3). This must result in material being removed from the surface under further loading and explain the origin of the released wear volume. Inter-granular cracks have already been described as one of the major causes of wear degradation on ceramic hip implants after in vitro tests on a non-standard hip-walking simulator that includes micro-separation [33,34,35] and on retrieved implants [15,36]. AFM images also revealed the presence of intra-granular cracked grains on the worn surface (Figure 4). As a matter of fact, this relevant point does highlight the release of wear debris that have smaller size and more irregular shape than the ones related to regular ceramic grains. As already mentioned by Zeng et al. [33] (alumina implant), such debris are prone to be furthermore trapped and piled into the microstructure (Figure 5). Afterwards the key point is around the biological responses of tissues and cells with this debris. Some investigations related to ceramic grains in contact with fibroblasts and osteoblasts have shown no toxicity [37]. Some tests may be investigated around small particles (debris) reactions with cells, tissues and animals in order to verify the absence of inflammation reaction.

The nanoindentation analysis confirms the role of shocks in the degradation and performance of the tested materials. We observed a significant variation of the mechanical properties (compared to the pristine values) only on the two samples subjected to shocks (Figure 8 and Figure 9). The hardness decreased from 8% (#SH6) to 9.5%(#SH9) (Figure 10). The Young’s modulus decreased slightly very close to the surface for the sample #SH9 (Table 2). We identify here two limits of this work that must be kept in mind for the analysis of these results and that must be balanced with high the number of nanoindentation tests (15):first, the number of specimens (*n* = 1) for each experimental condition; and, secondly, the fact that the relative results implied one single specimen (#PRIST) as a reference.

The two samples #SH9 and #SH6 exhibited the highest monoclinic phase content (35% and 40%, respectively, Figure 10). The phase transformation may however not be the direct cause of the decrease in *H* and *E*. This is supported by the fact that the mechanical properties of the sample #AUT remained unchanged while its monoclinic phase content increased (Figure 10); this sample showed a negligible surface degradation and no micro-cracks. Several authors [24,28,38,39] have already attributed such a decrease in *H* and *E* to micro-cracking on hydrothermally aged zirconia based ceramics. The AFM and DB-FIB analyses of the sample #SH9 also revealed here the presence of micro-cracks on the worn surface and in the first μm in depth. They may play a role about the mechanical properties deterioration and explain the dissemblance observed between the evolutions of *H* and *E*. The assessment of *E* is sensitive to the elastic field below the analyzed depth and the presence of micro-cracks on and near the surface has a low contribution in the whole field. The decrease in *E* is therefore lower than the one in H whose assessment is exclusively affected by the deterioration around the indenter. This observation may indicate that the damage is more extended in depth for the sample #SH9 compared with the sample #SH6 (Figure 9); this may explain the lower wear volume release for the latter [15]. A threshold in the intensity of shocks might exist between 6 and 9 kN and affect the material mechanical response and therefore the long-term structural integrity of the implant. Further investigations have to be carried out at other shock intensities in order to confirm the presence of this potential threshold.

ZTA composites have been developed as an alternative to alumina and zirconia based implants and aim at offering a wide range of application especially for young and active patients. The mechanical loads previously applied during shocks were high compared with that of in vivo environment [15] yet shocks created a damage that is restricted to the surface and to the first μm in depth. Comparative studies showed that alumina couplings exhibited wider wear stripes than ZTA ones [40] and that the crack network below the worn surfaces of alumina was denser than the one of a ZTA composite [38]. It is known that cracks generate mechanical stresses capable of enhancing the zirconia phase transformation; this phenomenon toughens zirconia-based materials by reducing the propagation of cracks [9]. The following scheme can therefore be suggested for the tested ZTA composite: (1) the high contact stresses during shocks are at the origin of the formation of micro-cracks within the wear stripes; (2) micro-cracks locally trigger the zirconia phase transformation that reduces their propagation in response; and (3) the remaining (limited) crack paths cause a decrease in the mechanical properties (*H* and *E*) in the damaged areas. The occurrence of the phase transformation mechanically induced within the stripes during shocks is therefore not detrimental for the tested ZTA, but rather a positive effect that limits the extension of the wear stripes.

## 5. Conclusions

This study presents a detailed analysis of the damage associated with in vitro tests able to simulate in vivo environment on ZTA hip joints. It highlights four key results:

1. The material exhibits an excellent resistance to hydrothermal ageing that does not affect its wear or mechanical performances on the surface.
2. Friction effects applied during hip-walking simulator test create non-homogenous wear features on the femoral head surface; the degree of wear degradation is low compared with that induced by shocks and raises the question of the relevance of the ISO 14242-1 standard wear test for ceramic hip implants.
3. Shocks under micro-separation release wear debris of various shapes and sizes through inter and intra-granular cracks and affect the implant hardness in the first μm in depth; we might see a threshold in the intensity of shocks between 6 and 9 kN, above which the Young’s modulus is affected.
4. The occurrence of the phase transformation is not detrimental when it is mechanically induced as a response of cracks formation; on the contrary, it toughens the material.

## Figures and Tables

**Table 1:** Details of the in vitro test and analyses previously performed on each femoral head (*n* = 1). The averaged values of *S*_a_ and *V*_m_ were measured on the head surface [14,15]. Analyses of wear stripe areas created by shocks in samples #SH6 and #SH9 are shown.

Head Name	In Vitro Test	*V*_m_ (%) Raman Spectroscopy	*S*_a_ (nm) 3D Profilometry
#PRIST	None	10 ± 1	<15
#AUT	360 h in autoclave	25 ± 3	<15
#SIM	6 M cycles hip-walking simulator	15 ± 4	<15
#SH6	1.5 M shocks at 6 kN	35 ± 3	>30
#SH9	1.5 M shocks at 9 kN	40 ± 2	>30

**Table 2:** Relative decrease (%) in hardness and Young’s modulus (*H*–*E*) with respect to the control-sample #PRIST at four specific depths (300, 600, 900 and 1200 nm).

Sample	300 nm	600 nm	900 nm	1200 nm
#AUT	2%–0%	1%–0%	1%–0%	1%–0%
#SIM	1%–0%	2%–1%	2%–0%	1%–0%
#SH6	15%–2%	9%–1%	6%–0%	4%–0%
#SH9	16%–4%	10%–4%	8%–2%	7%–1%
